# Identification of a novel, methylation-dependent, *RUNX2* regulatory region associated with osteoarthritis risk

**DOI:** 10.1093/hmg/ddy257

**Published:** 2018-08-10

**Authors:** Sarah J Rice, Guillaume Aubourg, Antony K Sorial, David Almarza, Maria Tselepi, David J Deehan, Louise N Reynard, John Loughlin

**Affiliations:** 1 Institute of Genetic Medicine, Newcastle University, International Centre for Life, NE1 3BZ, UK; 2 Newcastle University Teaching Hospitals NHS Trust, Freeman Hospital, High Heaton, NE1 7DN, UK

## Abstract

Osteoarthritis (OA) is a common, multifactorial and polygenic skeletal disease that, in its severest form, requires joint replacement surgery to restore mobility and to relieve chronic pain. Using tissues from the articulating joints of 260 patients with OA and a range of *in vitro* experiments, including CRISPR-Cas9, we have characterized an intergenic regulatory element. Here, genotype at an OA risk locus correlates with differential DNA methylation, with altered gene expression of both a transcriptional regulator (*RUNX2*), and a chromatin remodelling protein (*SUPT3H*). *RUNX2* is a strong candidate for OA susceptibility, with its encoded protein being essential for skeletogenesis and healthy joint function. The OA risk locus includes single nucleotide polymorphisms (SNPs) located within and flanking the differentially methylated region (DMR). The OA association SNP, rs10948172, demonstrates particularly strong correlation with methylation, and two intergenic SNPs falling within the DMR (rs62435998 and rs62435999) demonstrate genetic and epigenetic effects on the regulatory activity of this region. We therefore posit that the OA signal mediates its effect by modulating the methylation of the regulatory element, which then impacts on gene expression, with *RUNX2* being the principal target. Our study highlights the interplay between DNA methylation, OA genetic risk and the downstream regulation of genes critical to normal joint function.

## Introduction

Osteoarthritis (OA) is an age-related, complex disease characterized by the irreversible breakdown of articular cartilage, most commonly in the hip or knee (1). Under non-pathological conditions, articular cartilage facilitates joint function by minimizing friction and bearing the impact of mechanical loading (2). Loss of this cartilage is painful and, ultimately, debilitating. Current therapeutic intervention relies initially on symptomatic pain relief and replacement of the affected joint in end-stage disease (3). Genetic risk factors confer ∼50% of disease susceptibility and, to date, genome-wide association studies (GWAS) have reported up to 30 risk loci for OA (https://www.nature.com/articles/s41588-018-0079-y). Functional correlations have been established between genetic risk and epigenetic variation, principally via DNA methylation changes at CpG sites (4).

The common single nucleotide polymorphism (SNP) rs10948172 (A/G) has been associated with increased OA susceptibility in the UK GWAS arcOGEN study (*P* = 7 x 10^−8^) (5). This variant marks a region of ∼570 kb of high linkage disequilibrium (LD) at chromosome 6p21.1, containing the genes *SUPT3H* and *RUNX2*. These genes are transcribed in opposite directions and overlap at their 5′ ends. *SUPT3H* encodes a protein subunit of the transcription initiation complex STAGA, a histone acetyltransferase that preferentially acetylates histone 3 (H3) (6). *RUNX2* is a transcription factor essential for osteoblastic differentiation and skeletogenesis and has two main isoforms, encoded by distal (P1) and proximal (P2) promoter regions (7). Murine studies have shown regulation of *Runx2* gene expression by the promoter region of *Supt3h* during osteoblastic differentiation (8).

We have previously reported the results of a genome-wide methylation analysis of cartilage DNA using the Illumina Infinium Human Methylation 450 BeadChip array (450K array) (9,10). We observed correlation between genotype at rs10948172 and methylation at seven CpGs located close to *SUPT3H* and *RUNX2*, four 450K array CpGs (cg13979708, cg19254793, cg20913747 and cg18551225) and three CpGs that were captured using pyrosequencing (9). This cluster of seven CpGs are located 82 kb upstream of rs10948172, with the OA risk-conferring G allele of the SNP significantly correlating with reduced levels of methylation at each CpG (9). Together, these seven CpGs constitute a methylation quantitative trait locus (mQTL) and form a differentially methylated region (DMR), which will be referred to henceforth as the *SUPT3H/RUNX2*-DMR. Additional analysis revealed that this DMR was also present in two other joint tissues from OA patients: synovium and infrapatellar fat pad (9). None of the published epigenome-wide studies of OA cartilage have identified differential methylation at the four 450K array CpG sites in this DMR (10–14). These studies have considered methylation between OA and non-OA cartilage, intact and lesioned cartilage from the same joint and also amongst different regions of the tibial plateau. This indicates that differential methylation in this region is being driven by genotype and not disease state.

Two further GWAS reported that the SNP rs10948155 (T/C), which resides 89 kb upstream of rs10948172, correlates with the OA endophenotype of cartilage thickness (15,16). rs10948155 and rs10948172 flank the *SUPT3H/RUNX2-*DMR and have a pairwise r^2^ of 0.70 in Europeans. As such, they may be marking the same OA association signal*.* Furthermore, the *SUPT3H/RUNX2-*DMR contains two common SNPs, rs62435998 (C/T) and rs62435999 (T/G), that are in perfect LD with each other (r^2^=1.0) and in high LD with rs10948155 (r^2^=0.87) and rs10948172 (r^2^=0.80). In summary, there are two reported SNPs that correlate with OA phenotypes and that flank the *SUPT3H/RUNX2-*DMR and two SNPs that also correlate with these OA signals and reside within the DMR.

In this study, we set out to undertake a detailed molecular characterization of the *SUPT3H/RUNX2-*DMR and to investigate its regulatory effect on *SUPT3H* and *RUNX2*. We aimed to define the 5′ and 3′ limits of the DMR and to investigate the effect of SNP genotype upon methylation. We also searched for expression quantitative trait loci (eQTLs) at *SUPT3H* and *RUNX2*, and looked for correlations between mQTLs and eQTLs to establish the presence of methylation and eQTLs (meQTLs). Finally, using CRISPR-Cas9 and luciferase analysis, we determined the effect of DMR deletion on *SUPT3H* and *RUNX2* expression and of *in vitro* methylation on the activity of the DMR.

## Results

### *In silico* investigation into the *SUPT3H-RUNX2* DMR

**Figure 1:**
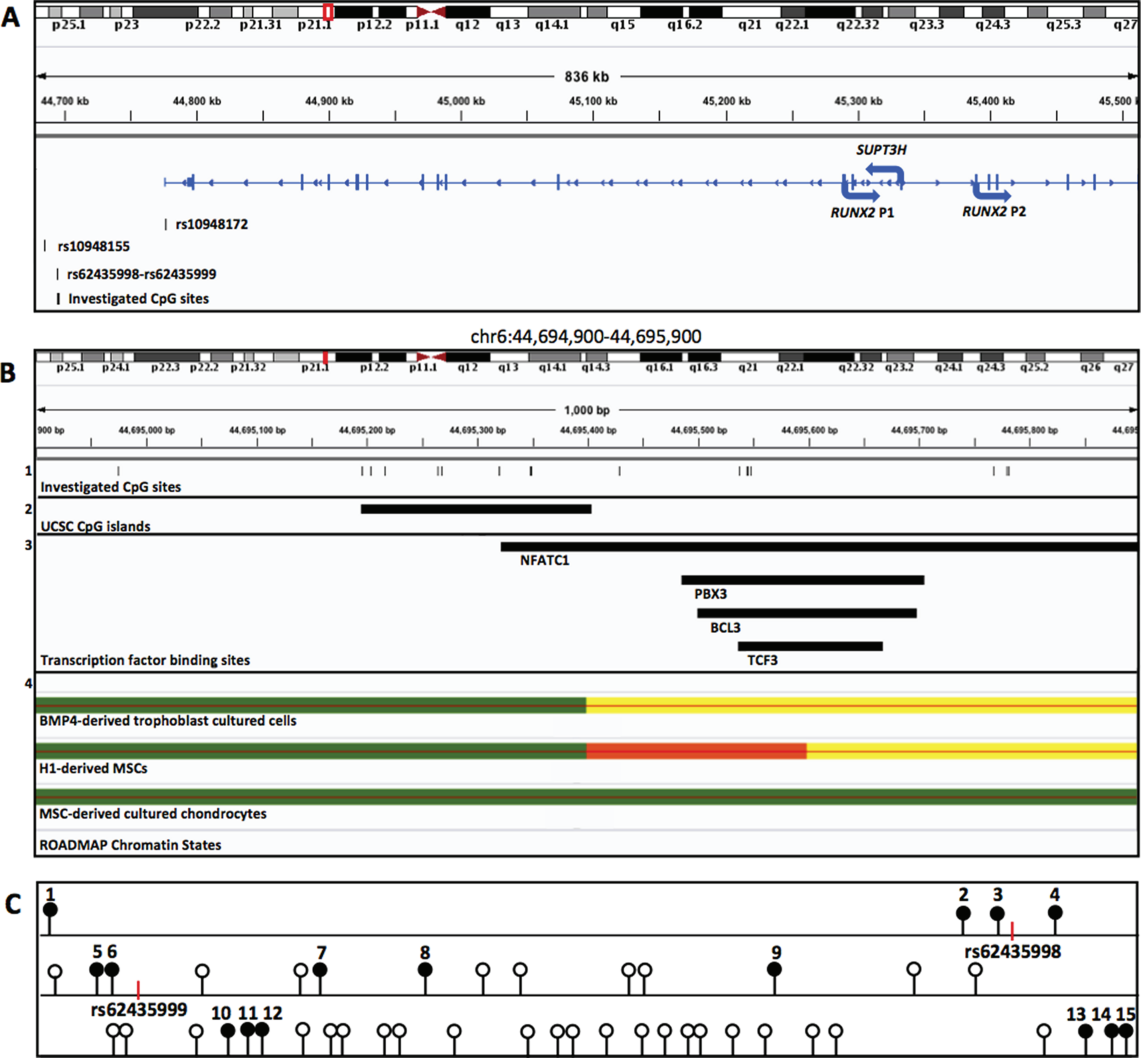
*In silico* investigation of the *SUPT3H/RUNX2*-DMR. (**A**) Chromosome 6p21.1 visualized using the Integrative Genomics Viewer displaying the location of the four SNPs and of the CpGs investigated relative to the *SUPT3H*, *RUNX2* (P1 isoform) and *RUNX2* (P2 isoform) transcripts. The region represented is indicated by the red box on the chromosome diagram. Arrows indicate the direction of transcription. (**B**) Zoomed-in image (red vertical line on chromosome) showing the location of the 15 CpGs analysed (1), the CpG island denoted on UCSC (2), the transcription factor binding sites (NFATC1, PBX3, BCL3 and TCF3) and (4) ROADMAP ChIP-seq data in three different cell types displaying peaks in this region. Yellow: weak enhancer, green: weak transcription, red: flanking transcription start site (TTS). Data are interpreted using the 18-state model. (**C**) Schematic representation of the 808 bp region in which the 15 investigated CpGs (solid black circles) reside. Open circles represent un-investigated CpGs. Vertical red lines mark the location of rs62435998 and rs62435999.

We previously identified seven CpG sites that correlate with rs10948172 genotype in OA patients (9). These reside within and on the south shore of a CpG island located 82 kb upstream of the OA association SNP rs10948172 (Fig. 1A and B). Our search of the University of California Santa Cruz (UCSC) Genome Browser and the Roadmap Epigenomics Project revealed that this DNAseI hypersensitive region is marked as a weak enhancer in multiple cell types, including MSCs and MSC-derived chondrocytes (Fig. 1B). This is indicated by the presence of weak H3K27ac peaks, along with signals for H3K36me3 in the 18-state model. Analysis of the 15-state model revealed peaks for H3K4me1, suggesting that this region is a poised enhancer in these cell types (17). Furthermore, this region is highly conserved in primates but is absent from the genome of quadrupedal mammals (data not shown).

Additional ENCODE ChIP-seq data revealed binding sites within the DMR for the transcription factors in the GM12872 lymphoblastoid cell line: NFATC1, PXB3, BCL3 and TCF3 (Fig. 1B).

### Physical limits of the *SUPT3H/*RUNX2-DMR

We next used pyrosequencing analysis to determine the 5′ and 3*′* physical limits of the DMR in OA patient DNA samples. We have previously reported that the DMR had a stronger correlation with rs10948172 genotype in synovium than cartilage (9) and we therefore used OA synovium DNA samples to determine its maximal limits. In our analysis, we included the four positive CpGs from the 450k array (CpGs 7–10 in Table S1) plus six CpGs upstream (CpG1–CpG6) and five CpGs downstream (CpG11–CpG15), spanning a region of 808 bp. All CpG sites within the investigated region are schematically represented in Figure 1C. The 15 CpG sites analysed in this study are highlighted.

**Figure 2:**
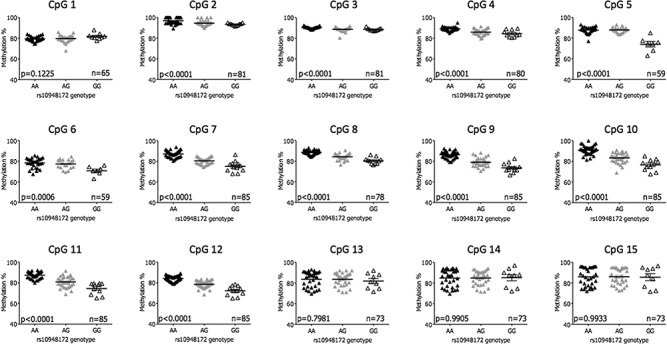
Definition of the 5′ and 3′ limits of the *SUPT3H/RUNX2*-DMR in OA synovium DNA**.** Graphs display the percentage methylation at each of the 15 CpG sites stratified by genotype at the OA association SNP rs10948172 (A/G). *P*-values were calculated using a one-way analysis of variance (ANOVA) with a Bonferonni post-test correction. The horizontal line represents the mean methylation and the standard error of the mean (SEM). n, the number of patients analysed per CpG.

A significant synovium mQTL (*P*<0.05) relative to genotype at rs10948172 was detectable at 11 of the 15 sites (Fig. 2). The 5′ limit of the DMR was determined exactly; an mQTL was detectable at CpG2 (*P*<0.0001) but not at CpG1 (*P*=0.12), with no further CpGs between the two. Analysis of the 3′ limit proved technically challenging due to the high density of CpG sites in this region (Fig. 1C). However, the three CpGs captured by assay 8 (CpG13–CpG15) showed no significant mQTL (*P*>0.05, Fig. 2), and as such the DMR extends from CpG2 up to, but not including, CpG13, a region of 572 bp.

We subsequently assessed the limits of the DMR in cartilage and fat pad DNA (Figs. S1 and S2), again relative to genotype at rs10948172. In these tissues, no significant mQTL was detected at CpG2, which informed us that we had defined the 5′ limit of the DMR. Therefore, analysis of CpG1 was not undertaken in these tissues. In total, we analysed 14 of the 15 CpGs that have been studied in our synovium DNAs, CpG2–CpG15. A significant mQTL was observed at CpGs 6–12 in cartilage and CpGs 3–12 (with the exception of CpG 6) in fat pad.

Finally, we investigated the existence of the *SUPT3H/RUNX2*-DMR in trabecular bone DNA from patients with OA, a tissue not previously studied. Due to the relatively modest sample size of up to 31 bone samples, only two homozygotes for the OA risk-conferring G allele of rs10948172 were identified (Table S2). We therefore measured the effect of the risk allele by comparing AA homozygotes to those carrying one or two copies of the risk allele (AG/GG) (Fig. S3). Two of the 14 CpGs showed a significant mQTL: CpG 7 and CpG 11.

In summary, the DMR was active across four joint tissues from patients with OA and was narrowed to a maximum interval of 572 bp in synovium.

### The effect of genotype upon methylation within the *SUPT3H/*RUNX2-DMR

We subsequently determined the extent to which genotype at the OA association SNPs rs10948172 and rs10948155, and at the DMR SNPs rs62435998 and rs62435999, influences methylation in this region. Since rs62435998 and rs62435999 are in perfect LD, we only genotyped one of these SNPs (rs62435998).

**Figure 3:**
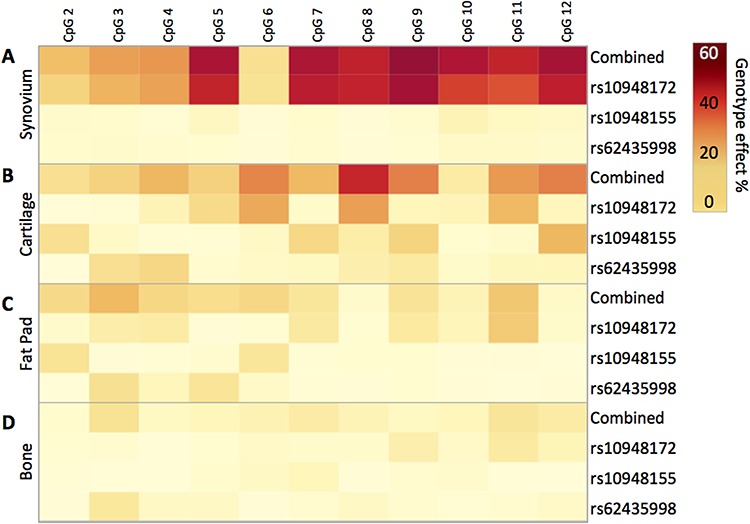
The influence of genotype upon methylation at the *SUPT3H/RUNX2-*DMR. Heat map displaying the percentage effect of genotype at rs10948172, rs10948155 and rs62435998, plus all three SNPs combined, at CpG2 to CpG12 in the following: (**A**) synovium (n = 59 patients), (**B**) cartilage (n = 30 patients), (**C**) fat pad (n = 44 patients) and (**D**) bone (n = 25 patients). The relative contribution of each SNP was calculated using the log worth and incremental R^2^ statistics. The incremental improvement in R^2^ values capturing explained variability was then plotted. Statistical analyses were performed with the aid of SAS JMP Statistical Data Visualization Software.

Using samples that had a complete data set of matched methylation and genotype data, we constructed a linear model of the percentage methylation at the individual sites assessing the relative contribution of each SNP to differential methylation. We then constructed a heat map of the percentage genotypic effect at each CpG site in all four investigated OA joint tissues (Fig. 3). In total, we had complete data available for 59 synovium, 30 cartilage, 44 fat pad and 25 bone DNA samples.

The strongest mQTL was detected in synovium, followed by cartilage, fat pad and then bone. In synovium, the strongest effect was at CpG9, with genotype at all three SNPs combined accounting for 65.79% of variability in methylation. The vast majority of this genotypic effect was explained by genotype at rs10948172 (63.08%), with genotype at rs10948155 and rs62435998 providing modest contributions (1.73% and 0.98%, respectively). A broadly similar effect was observed at all CpG sites analysed in synovium, with the exception being CpG6, where only a weak mQTL was detected. These data indicate that in synovium, genotype at rs10948155 and rs62435998 does not function in coordination with rs10948172 to provide a haplotypic effect.

In cartilage, the strongest effect was at CpG8, with genotype at the three SNPs combined accounting for 54.62% of variability. Again, rs10948172 explained the majority of this effect, with a contribution of 33.99%. However, here we observed more substantive contributions from rs10948155 and rs62435998. In fat pad and bone, where weaker mQTLs were detected, less of the variation in methylation was explained by genotype at all three SNPs, with measured values of up to 26.86% in fat pad and 15.73% in bone (Fig. 3).

Overall, this analysis highlighted that the arcOGEN SNP rs10948172 is the principal driver of the observed differential methylation, with particularly strong effects in both synovium and cartilage.

### Allelic expression imbalance analysis of *SUPT3H* and *RUNX2*

**Figure 4:**
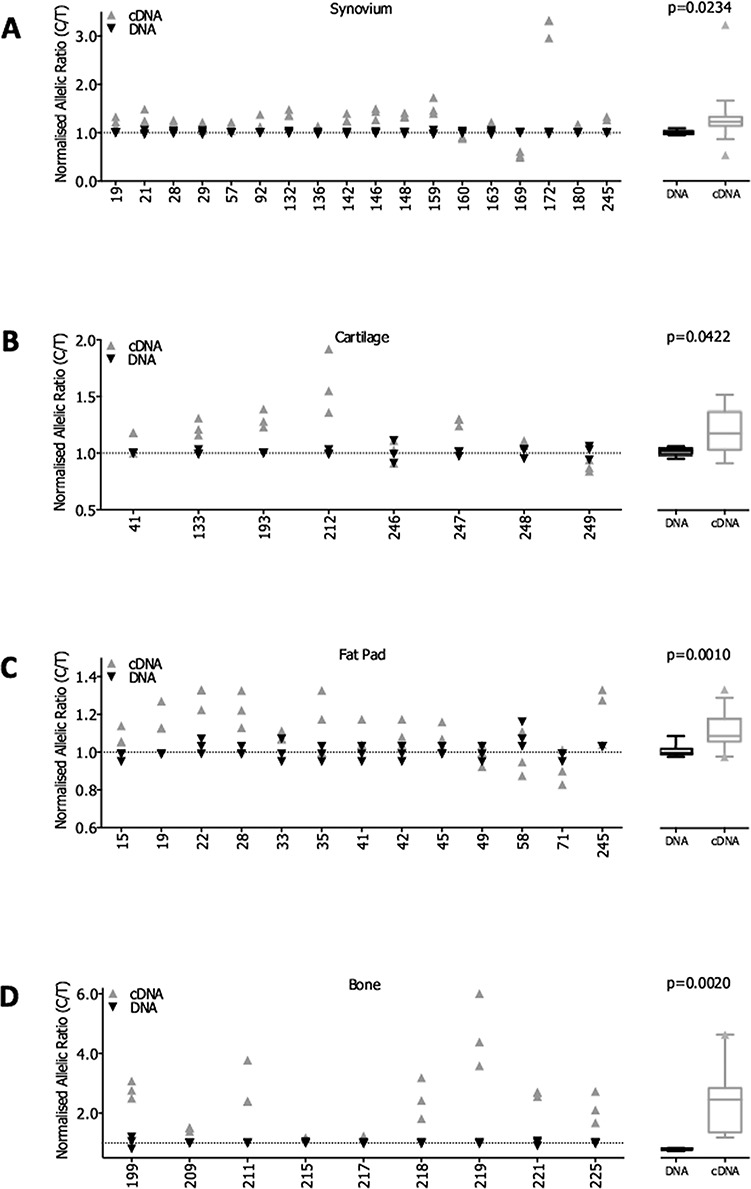
AEI analysis of *SUPT3H* using the transcript SNP rs529125. AEI was carried out in OA patient samples from the following tissues: (**A**) synovium (n = 18 patients), (**B**) cartilage (n = 8 patients), (**C**) fat pad (n = 13 patients) and (**D**) bone (n = 9 patients). Data for individual patients are plotted as the ratio of the risk C allele divided by the non-risk T allele, with the cDNA ratio normalized to that measured in the DNA of the same patient sample. A ratio > 1 indicates an increased expression of the risk C allele. Each patient DNA and cDNA sample was analysed in triplicate. X-axis lists the patient IDs. The mean DNA and cDNA values for all patients combined in each tissue are displayed as a box plot denoting the median value, along with the 25^th^ to 75^th^ percentiles. Whiskers represent the 10^th^ and 90^th^ percentiles, and any outliers are displayed. *P*-values were calculated using a two-tailed paired *t*-test.

We next used allelic expression imbalance (AEI) analysis to measure rs10948172 eQTLs operating on *SUPT3H* and *RUNX2*. In all four joint tissues, we identified an increase in expression of the C allele of the *SUPT3H* transcript SNP rs529125, which is equivalent to the OA risk-conferring G allele of rs10948172 (Fig. 4). The greatest imbalance was detected in bone samples, with a mean increase of 2.2-fold (*P*=0.002, Fig. 4D). In the other tissues, the mean increases were 1.17-fold in synovium (*P*=0.023, Fig. 4A), 1.15-fold in cartilage (*P*=0.0422, Fig. 4B) and 1.11-fold in fat pad (*P*=0.001, Fig. 4C). A *SUPT3H* eQTL operating in association with rs10948172 has been reported in 10 different tissues on the genotype-tissue expression (GTEx) browser (18).

**Figure 5:**
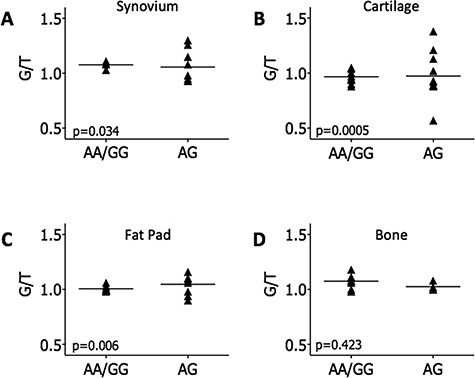
AEI analysis of *RUNX2* using the transcript SNP rs1200428**.** AEI was carried out in rs1200428 heterozygous OA patient samples from the following tissues: (**A**) synovium (n = 13 patients), (**B**) cartilage (n = 22 patients), (**C**) fat pad (n = 17 patients) and (**D)** bone (n = 12 patients). The ratio of G/T alleles was determined in patient DNA and cDNA and each cDNA ratio was normalized to that measured in the DNA of the same patient sample. These data were stratified by genotype at the association SNP, rs10948172. Each patient DNA and cDNA sample was analysed in triplicate and the mean cDNA ratio per patient is plotted. *P*-values were calculated using an unpaired *t*-test of variances. Horizontal lines represent the mean values across patients.

*RUNX2* AEI was measured in all four joint tissues. Here, the variance in compound heterozygotes was compared to that measured in patients who were homozygotic at the OA association SNP. Significant AEI was detected in synovium (*P*=0.034, Fig. 5A), cartilage (*P*=0.0005, Fig. 5B) and fat pad (*P*=0.006, Fig. 5C) but not in bone samples (Fig. 5D). Due to the linkage equilibrium between rs10948172 and the *RUNX2* transcript SNP rs1200428 (r^2^=0.005), it is not possible to determine the direction of the *RUNX2* AEI.

In summary, the AEI analysis demonstrated the presence of an rs10948172 eQTL operating on both *SUPT3H* and *RUNX2* in multiple joint tissues.

### The impact of the mQTL upon gene expression of *SUPT3H* and *RUNX2*

Following the characterization of the mQTL at the *SUPT3H/RUNX2* locus and the identification of eQTLs operating on both *SUPT3H* and *RUNX2*, we next set out to assess the presence of an meQTL. To achieve this, we stratified the methylation values at the CpG sites by the AEI ratios in patient samples for which matched methylation and AEI data were available. We focused on the 11 CpGs that generated evidence of mQTLs in our earlier analysis, CpG2–CpG12 (Fig. 2 and Figs. S1–S3).

For *SUPT3H*, we had observed AEI in all four tissues (Fig. 4), but we did not observe any significant correlations between CpG methylation and AEI ratio in any of the tissues when they were analysed separately (data not shown).

**Figure 6:**
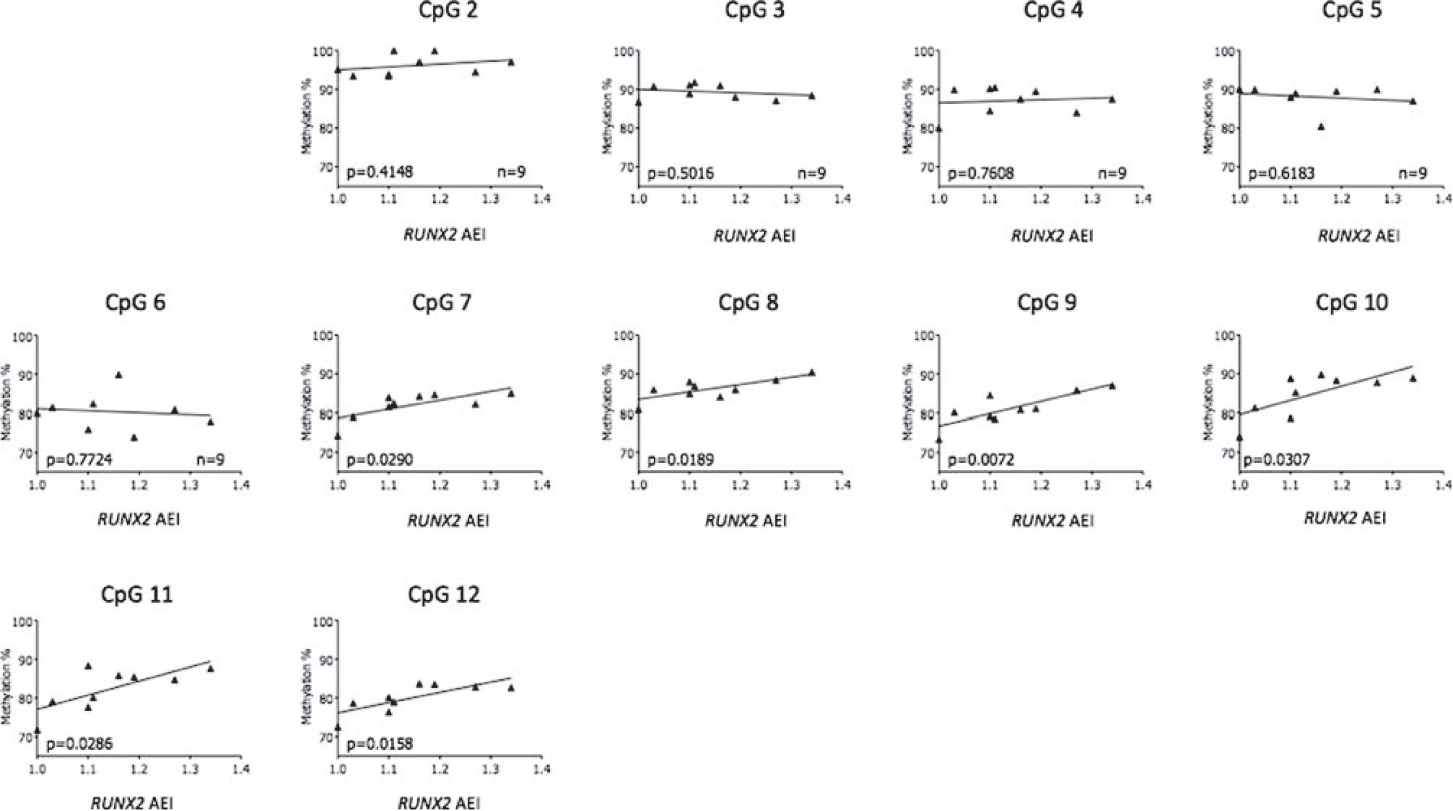
Correlation between AEI at *RUNX2* and methylation at the *SUPT3H/RUNX2-DMR*. Normalized cDNA allelic ratios of *RUNX2* expression in synovium were plotted against the respective DNA methylation values at CpG2 through to CpG12. *P*-values were calculated by linear regression analysis. n, the number of patients analysed per CpG.

Due to the linkage equilibrium between the *RUNX2* transcript SNP rs1200428 and rs10948172, it was necessary to assume that all patient samples demonstrated AEI in the same direction for this gene. Therefore, those samples with AEI ratios <1 were inverted to provide a unidirectional imbalance in allelic expression. The meQTL analysis was then carried out in synovium, cartilage and fat pad samples as they were the three tissues in which *RUNX2* AEI was detected (Fig. 5). Significant correlation was observed in synovium at six CpGs (CpG7–CpG12) (Fig. 6). In fat pad, a significant meQTL was observed at CpG4 (*P*=0.009), with CpG12 approaching significance (Fig. S4A). No significant meQTL was detected in cartilage (Fig. S4B). The absence of LD between rs1200428 and rs10948172 precludes us from determining the direction of the relationship between methylation and allelic expression of *RUNX2*.

Overall, this analysis revealed a significant correlation with DMR methylation levels and the level of AEI of *RUNX2* in synovium and fat pad but not cartilage. We observed no correlation between methylation and *SUPT3H* AEI in any tissue analysed*,* where the genetic and epigenetic effects appear to be functioning independently*.*

### Functional analysis of *SUPT3H/RUNX2*-DMR using CRISPR-Cas9

**Figure 7:**
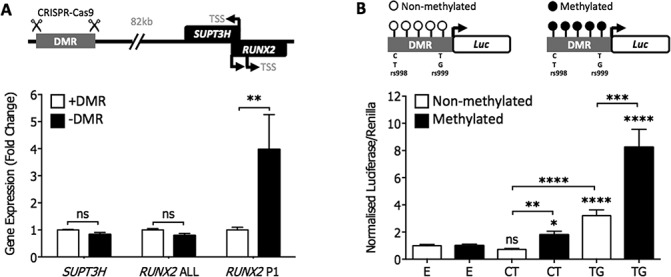
*In vitro* modulation of the *SUPT3H/RUNX2*-DMR. **(A)** CRISPR-Cas9 deletion of the DMR in the chondrocyte cell line Tc28a2 (TSS). Expression of *SUPT3H, RUNX2* ALL (both isoforms) and *RUNX2* P1 (long isoform) was measured by *RT-qPCR*. The graph represents the mean (+/− SEM) fold change of each gene/isoform following deletion of the DMR (−DMR) compared to control cells without deletion (+DMR). Each bar represents six biological repeat experiments. Statistical significance was determined by two-way ANOVA. **, *P* < 0.005; ns, not significant, *P* > 0.05. (**B)** Luciferase reporter analysis of DMR methylation in the chondrosarcoma cell line SW1353. Mean luciferase activity (21 technical repeats) of the DMR normalized against a control empty non-methylated or empty methylated vector. E is the empty vector control. CT denotes the non-risk conferring haplotype at rs62435998-rs62435999. TG denotes the risk-conferring haplotype at s62435998-rs62435999. Error bars represent the SEM. *P*-values were calculated using a Mann–Whitney two-tailed exact test and are compared to the relevant non-methylated or methylated empty vector control, unless otherwise indicated. *, *P* < 0.05; **, *P* < 0.01; ***, *P* < 0.001; ****, *P* < 0.0001.

We next assessed the effect that deletion of the DMR had on *SUPT3H* and *RUNX2* expression. Using the chondrocyte cell line Tc28a2 and the CRISPR-Cas9 genome editing system, we deleted a 600 bp region that encompassed the 572 bp DMR that we had defined. This had no effect on *SUPT3H* expression or on the combined expression of both P1 and P2 *RUNX2* isoforms (measured using the *RUNX2* ALL assay). However, when *RUNX2* P1 was measured alone, a mean 3.9-fold increase in gene expression was observed (*P*<0.005, Fig. 7A). These *in vitro* data support the DMR as a regulator of *RUNX2* expression.

### Relationship between DMR methylation and its function

Finally, we investigated the effect of *in vitro* methylation on the DMR. In this analysis, we also investigated any effect of genotype at the two SNPs located within the DMR, rs62435998 and rs62435999. The common CT haplotype of rs62435998-rs6243599 corresponds to the non-risk alleles of rs10948172 (A) and rs10948155 (T), whilst the TG haplotype corresponds to the risk-conferring alleles of rs10948172 (G) and rs10948155 (C). Both haplotypes of the DMR were separately cloned into the pCpGL luciferase vector (19) and analysed with and without *in vitro* methylation. This vector contains no CpG sites, allowing the effects of DMR methylation on luciferase activity to be determined independent of any effects conferred by vector methylation. The vectors were then transfected into the chondrosarcoma cell line SW1353.

Without methylation, the non-risk CT construct showed no difference in activity relative to the empty pCpGL vector, whereas the risk-conferring TG construct showed a 3.2-fold increase in luciferase activity (*P*<0.0001) (Fig. 7B). With methylation, both constructs showed a significant increase in activity: CT, 1.79-fold above control, *P*<0.01; TG, 8.24-fold above control, *P*<0.001. These results demonstrate that the *SUPT3H/RUNX2*-DMR displays transcriptional regulatory activity in SW1353 cells and that methylation of the region enhances this effect. The data also indicate that the risk haplotype is a stronger regulator than the non-risk haplotype.

## Discussion

Using DNA isolated from the joint tissues of patients with OA, we were able to narrow the *SUPT3H/RUNX2*-DMR to a 572 bp interval. To our knowledge, this is the first published study into the physical limits of an OA-associated mQTL, reported here in multiple tissues. The presence of this mQTL in multiple tissues of the articulating joint highlights the joint-wide pathology of OA. *In silico* analysis of publicly available data revealed the region as having transcriptional regulatory properties. ChIP-seq data reveal that several cartilage-expressed transcription factors bind to this region in the GM12872 cell line. Stratification by genotype at OA-associated SNPs highlighted the arcOGEN OA SNP rs10948172 as showing particularly strong correlation with the methylation at the DMR. This was an important observation as it suggests a mechanism by which this OA risk-conferring signal mediates its effect, namely modulating methylation at the DMR, which then alters gene expression at the allelic level (Fig. S5). To determine whether this was plausible, we first assessed whether *SUPT3H* and *RUNX2* were subject to an rs10948172 eQTL in OA tissues. Having determined that this was the case for both genes, we were then able to show that *RUNX2* AEI correlated with methylation in synovium and fat pad, revealing an meQTL. We next directly demonstrated that DMR was a transcriptional regulator by deleting it in the Tc28a2 cell line, observing altered expression of the P1 isoform of *RUNX2*. This isoform is predominantly expressed in mesenchymal stem cells through to chondrocyte progenitor cells and in mature osteoblasts (20–22). We finally undertook *in vitro* analysis of the DMR and we were able to show that both methylation and haplotype directly regulated gene transcription. This supports the functional mechanism posited in Supplementary Figure S5, in that it clearly demonstrates that methylation of the DMR alters gene expression. In this final experiment, we included genotype at the two common DMR SNPs rs62435998 and rs6243599 as variables. We noted that the OA risk-conferring haplotype of these SNPs resulted in increased expression relative to the non-risk haplotype, with and without DMR methylation. This is a key observation as it highlights that these two polymorphisms have both genetic and epigenetic effects on the activity of the DMR. It also implies that the common OA-associated SNPs at the *SUPT3H/RUNX2* locus may be acting in concert to modulate OA risk.

A search of the GTEx portal (18) reveals that there are at least 10 tissues in which *SUPT3H* eQTLs have been reported that correlate with rs10948172 genotype. These are present in a range of tissues including brain, tibial nerve, testis and spleen. In each case, the OA risk-conferring G allele correlates with higher levels of *SUPT3H* expression, a pattern that we observed in OA joint tissues. As far as we are aware, there are no previous reports of the *RUNX2* eQTL detected in this study, indicating that this effect may be specific to certain tissues of the articulating joint.

In our meQTL analysis, we noted that the methylation expression effect was only present for *RUNX2*. This, combined with the fact that deletion of the DMR altered *RUNX2* but not *SUPT3H* expression, leads us to conclude that *RUNX2* is the principal target of regulation by the DMR. The meQTL was identified at six CpG sites within the DMR (CpG7–12). All of these CpG sites fall within the binding site of transcription factor NFATC1, with three of these CpGs (CpG10–12) also being located within the binding site of transcription factors BCL3, PBX3 and TCF3. This potentially highlights NFATC1, a protein that has previously been linked to OA pathophysiology (23), as the primary transcription factor operating at the *SUPT3H/RUNX2-*DMR.

Genetic effects are operating at this locus in association with *SUPT3H.* A potential role for this histone remodelling complex subunit in the progression of OA still remains, yet the underlying mechanism is unclear. The RUNX2 transcription factor has been extensively studied in humans and in animal models. It is essential for skeletogenesis and osteoblast differentiation, with knockout mice lacking endochondral ossification (24). Furthermore, an increase in *RUNX2* expression has been well established in OA cartilage, where the transcription factor is associated with chondrocyte hypertrophy and an increase in expression of the collagenase MMP13 (25–27). In a recent study, it was reported that deletion of the gene attenuates the progression of surgically induced OA in mice (28). Comprehensive knowledge of the regulation of *RUNX2* is therefore crucial to understanding both normal and pathological development and maintenance of the skeletal system.

In summary, we have undertaken a detailed characterization of the *SUPT3H/RUNX2*-DMR and established that it is a novel regulator of gene expression. Common polymorphisms that are associated with OA correlate with differential methylation of the DMR and altered expression of *RUNX2*. We propose that this is the likely mechanism by which the OA association signal at this locus operates. This opens the opportunity for further investigation, including potential intervention, by epigenetic modulation of the DMR to fine-tune *RUNX2* expression.

## Materials and Methods

### Patients

Tissue samples were obtained with informed consent from patients undergoing elective total joint replacement surgery for primary hip or knee OA, as described previously (29). Ethical approval was granted by the Newcastle and North Tyneside Research Ethics Committee (REC reference number 14/NE/1212). In total, we collected and analysed tissue samples from a total of 260 patients. Further details are outlined in Supplementary Table S2.

Tissue was processed as previously described (30). Nucleic acids were extracted from cartilage and trabecular bone samples using TRIzol reagent (Life Technologies) and from synovium and infrapatellar fat pad using the EZNA DNA/RNA Isolation kit (Omega Bio-Tek) according to the manufacturers’ instructions. For genotyping and AEI analysis, DNA was used directly. For methylation analysis, 500 ng DNA was bisulphite converted using the EZ DNA methylation kit (Zymo Research). RNA was quantified by Nanodrop (Thermo Fisher) and 1 μg (250 ng/μl) was treated with Turbo DNase (Thermo Fisher) prior to a 20 μl reverse transcription reaction with SuperScript First-Strand cDNA synthesis kit (Invitrogen). The cDNA product was then used for AEI and quantitative real-time PCR (RT-qPCR) analysis.

### Genotyping

Genotyping at rs10948172 was completed by pyrosequencing at proxy SNP rs529125 (r^2^=1.0). rs10948155 was genotyped directly by pyrosequencing. Genotype at rs62435998 was determined by a restriction fragment length polymorphism (RFLP) assay using the enzyme *HaeIII*, which cuts at the C allele of the SNP. Both pyrosequencing RFLP analysis were carried out in patient DNA samples following polymerase chain reaction (PCR) amplification of the region encompassing the SNP (Table S2). For pyrosequencing, PCR products were analysed using the PyroMark Q24 Advanced platform (Qiagen) along with the appropriate sequencing primer (Table S3) and the manufacturer’s recommended reagents.

### Methylation analysis

DNA methylation was quantified by pyrosequencing. Assays were designed to target specific CpG sites of interest using the PyroMark Assay Design Software (Qiagen). Eight assays were designed that each captured between 1 and 3 CpGs, with a total of 15 CpGs analysed (Table S3). These included the four positive CpGs from our 450k array analysis (cg13979708, cg19254793, cg20913747 and cg18551225; CpGs 7–10 in Table S1). Pyrosequencing was performed using the PyroMark Q24 platform as described previously (9). To determine the effect of genotype at the SNPs of interest upon methylation, we constructed a linear model of the percentage methylation at individual CpG sites, assessing the relative contribution of each SNP using the log worth and incremental R^2^ statistics. To visualize the SNPs most closely associated with methylation at a given site, the incremental improvement in R^2^ values capturing explained variability was expressed as a percentage effect and plotted as a heat map, both for the individual SNP data and for all of the SNPs combined. Statistical analyses were performed with the aid of SAS JMP Statistical Data Visualization Software.

### AEI

AEI was used to determine the presence of rs10948172 eQTLs for *SUPT3H* and *RUNX2*. Using a transcript SNP for each gene, pyrosequencing was used to determine the percentage of each allele in DNA and RNA, as described previously (31). For *SUPT3H*, we used rs529125 (T/C), which is in perfect LD with rs10948172; the OA risk-conferring G allele of rs10948172 corresponds to the C allele of rs529125. There are no *RUNX2* transcript SNPs in LD with rs10948172 and we therefore selected an SNP with a high minor allele frequency (MAF) to enable us to identify compound heterozygotes. We chose rs1200428 (G/T, MAF = 0.23; r^2^ = 0.005 with rs10948172). Due to the lack of LD between rs1200428 and rs10948172, normalized heterozygote allelic ratios were calculated and stratified by rs10948172 genotype as previously described (32,33). The PCR and sequencing primers are listed in Table S4. For each cDNA and DNA sample, PCR reactions were formed in triplicate, with samples being excluded from the analysis if the values between the PCR replicates differed by >5%.

### CRISPR-Cas9

Guide RNA (gRNA) sequences targeting upstream (5′CAAAACTTCTAGTCCCTAGA3′) and downstream (5′GAGACCGCGCCACAGGAGGA3′) of the *SUPT3H/RUNX2*-DMR were designed using the Optimized CRISPR Design Tool (crispr.mit.edu/). Single-stranded DNA oligonucleotides (Sigma Aldrich) containing the desired gRNA sequence along with the *BbsI* recognition motif were annealed with the reverse complementary strand (95°C–25°C, Δ-6°C/min). The double-stranded DNA oligonucleotides were then ligated into the *BbsI* linearized CRISPR-Cas9 vector, PX462, using T4 ligase (Invitrogen) overnight at 16°C. Cells from the human chondrocyte cell line Tc28a2 were grown in monolayer culture as previously described (34) to 70–80% confluence. For nucleofection, 10^6^ cells per biological replicate were incubated with 5 μg of plasmid DNA using the manufacturer-recommended Cell Line 4D-Nucleofector X Kit in combination with the 4D-Nucleofector System (Lonza).

Following nucleofection, cell monolayers were grown in each well of a six well plate and nucleofected cells were selected with puromycin after 24 h. Following expansion of cells into T25 culture flasks, nucleic acids were extracted using the EZNA DNA/RNA Isolation kit (Omega Bio-Tek) according to the manufacturer’s protocol. Deletion of the target region was confirmed and quantified using endpoint PCR and Sanger sequencing.

### Quantitative gene expression

Gene expression was quantified using TaqMan chemistry. Pre-designed TaqMan assays (Integrated DNA Technologies) were used to measure the expression of the target genes *SUPT3H* (Hs.PT.58.3510503) and *RUNX2*. For *RUNX2*, two assays were used: one targeting exons 6–7, measuring both the isoforms of the gene (Hs.PT.56a.19568141, termed *RUNX2* ALL) and one targeting exons 1–2, therefore only detecting the P1 isoform (Hs.PT.56a.23056352.g). It was not possible to measure the expression of the P2 isoform alone due to the sequence identity between the short isoform and exons 3–8. Expression was assessed relative to the expression of three housekeeping genes: *18S, GAPDH* and *HPRT1* using the 2^-Δct^ method as previously described (9).

### Luciferase reporter assays

A 654 bp region (chr6:44,695,068-44,695,721; hg19) containing the *SUPT3H/RUNX2*-DMR was cloned into the multiple cloning site of the CpG-free luciferase vector, pCpGL (InvivoGen) using the *PstI* and *BamHI* restriction sites (forward primer: 5′GGGGCTGCAGAGCTTCAAAACAGGAAAACCAAG3′; reverse primer: 5′GGGGCCATGGCACTGTTGAGCAAATCATCAGG3′). The region was amplified from a pool of genomic blood DNA and colonies were isolated and Sanger sequenced to identify clones with the major (CT) haplotype and clones with the minor (TG) haplotype of rs62435998-rs62435999. Plasmids were *in vitro* methylated using *M.SssI* (NEB) as previously described (35) and complete methylation was confirmed by *HpaII* restriction digest. For each well of a 96 well plate, 6000 cells from the chondrosarcoma cell line SW1353 were seeded 24 h prior to transfection. Cells were transfected with the relevant pCpGL luciferase vector construct (100 ng) and pRL-TK Renilla vector (1.5 ng) using Fugene HD transfection reagent (Promega) according to the manufacturer’s protocol. After 24 h, cells were lysed and luciferase reporter assays were performed (29).

## Supplementary Material

Supplementary Data

## Data Availability

All data are available from the corresponding author upon request.

## References

[1] Cross M., Smith E., Hoy D., Nolte S., Ackerman I., Fransen M., Bridgett L., Williams S., Guillemin F., Hill C.L. et al. (2014) The global burden of hip and knee osteoarthritis: estimates from the global burden of disease 2010 study. Ann. Rheum. Dis., 73, 1323–1330. 24553908 10.1136/annrheumdis-2013-204763

[2] Loeser R.F., Goldring S.R., Scanzello C.R. and Goldring M.B. (2012) Osteoarthritis: a disease of the joint as an organ. Arthritis Rheum., 64, 1697–1707. 22392533 10.1002/art.34453 PMC3366018

[3] Dieppe P.A. and Lohmander L.S. (2005) Pathogenesis and management of pain in osteoarthritis. Lancet, 365, 965–973. 15766999 10.1016/S0140-6736(05)71086-2

[4] Loughlin J. and Reynard L.N. (2015) Osteoarthritis: epigenetics of articular cartilage in knee and hip OA. Nat. Rev. Rheumatol., 11, 6–7. 25366188 10.1038/nrrheum.2014.189

[5] arcOGEN Consortium, Zeggini E., Panoutsopoulou K., Southam L., Rayner N.W., Day-Williams A.G., Lopes M.C., Boraska V., Esko T., Evangelou E. et al. (2012) Identification of new susceptibility loci for osteoarthritis (arcOGEN): a genome-wide association study. Lancet, 380, 815–823. 22763110 10.1016/S0140-6736(12)60681-3 PMC3443899

[6] Martinez E., Palhan V.B., Tjernberg A., Lymar E.S., Gamper A.M., Kundu T.K., Chait B.T. and Roeder R.G. (2001) Human STAGA complex is a chromatin-acetylating transcription coactivator that interacts with pre-mRNA splicing and DNA damage-binding factors *in vivo*. Mol. Cell Biol., 21, 6782–6795. 11564863 10.1128/MCB.21.20.6782-6795.2001 PMC99856

[7] Stock M. and Otto F. (2005) Control of *RUNX2* isoform expression: the role of promoters and enhancers. J. Cell Biochem., 95, 506–517. 15838892 10.1002/jcb.20471

[8] Barutcu A.R., Tai P.W., Wu H., Gordon J.A., Whitfield T.W., Dobson J.R., Imbalzano A.N., Lian J.B., Wijnen A.J. van, Stein J.L. et al. (2014) The bone-specific *Runx2*-P1 promoter displays conserved three-dimensional chromatin structure with the syntenic *Supt3h* promoter. Nucleic Acids Res., 42, 10360–10372. 25120271 10.1093/nar/gku712 PMC4176362

[9] Rushton M.D., Reynard L.N., Young D.A., Shepherd C., Aubourg G., Gee F., Darlay R., Deehan D., Cordell H.J. and Loughlin J. (2015) Methylation quantitative trait locus analysis of osteoarthritis links epigenetics with genetic risk. Hum. Mol. Genet., 24, 7432–7444. 26464490 10.1093/hmg/ddv433 PMC4664171

[10] Rushton M.D., Reynard L.N., Barter M.J., Refaie R., Rankin K.S., Young D.A. and Loughlin J. (2014) Characterization of the cartilage DNA methylome in knee and hip osteoarthritis. Arthritis Rheumatol., 66, 2450–2460. 24838673 10.1002/art.38713 PMC4314681

[11] Hollander W. den, Ramos Y.F., Bos S.D., Bomer N., Breggen R. van der, Lakenberg N., Dijcker W.J. de, Duijnisveld B.J., Slagboom P.E., Nelissen R.G. et al. *,* (2014) Knee and hip articular cartilage have distinct epigenomic landscapes: implications for future cartilage regeneration approaches. Ann. Rheum. Dis. 73**,** 2208–2212. 25261579 10.1136/annrheumdis-2014-205980

[12] Jeffries M.A., Donica M., Baker L.W., Stevenson M.E., Annan A.C., Humphrey M.B., James J.A. and Sawalha A.H. (2014) Genome-wide DNA methylation study identifies significant epigenomic changes in osteoarthritic cartilage. Arthritis Rheumatol., 66, 2804–2815. 24980887 10.1002/art.38762

[13] Alvarez-Garcia O., Fisch K.M., Wineinger N.E., AkagiL R., Saito M., Sasho T., Su A.I. and Lotz M.K. (2016) Increased DNA methylation and reduced expression of transcription factors in human osteoarthritis cartilage. Arthritis Rheumatol., 68, 1876–1886. 26881698 10.1002/art.39643 PMC4963260

[14] Zhang Y., Fukui N., Yahata M., Katsuragawa Y., Tashiro T., Ikegawa S. and Michael Lee M.T. (2016) Genome-wide DNA methylation profile implicates potential cartilage regeneration at the late stage of knee osteoarthritis. Osteoarthritis Cartilage., 24, 835–843. 26746145 10.1016/j.joca.2015.12.013

[15] Castano-Betancourt M.C., Cailotto F., Kerkhof H.J., Cornelis F.M., Doherty S.A., Hart D.J., Hofman A., Luyten F.P., Maciewicz R.A., Mangino M. et al. (2012) Genome-wide association and functional studies identify the *DOT1L* gene to be involved in cartilage thickness and hip osteoarthritis. Proc. Natl. Acad. Sci. USA., 109, 8218–8223. 22566624 10.1073/pnas.1119899109 PMC3361426

[16] Castano-Betancourt M.C., Evans D.S., Ramos Y.F., Boer C.G., Metrustry S., Liu Y., Hollander W. den, Rooij J. van, Kraus V.B., Yau M.S. et al. (2016) Novel genetic variants for cartilage thickness and hip osteoarthritis. PLoS Genet., 12, e1006260. 10.1371/journal.pgen.1006260 PMC5049763 27701424

[17] Creyghton M.P., Cheng A.W., Welstead G.G., Kooistra T., Carey B.W., Steine E.J., Hanna J., Lodato M.A., Frampton G.M., Sharp P.A. et al. (2010) Histone H3K27ac separates active from poised enhancers and predicts developmental state. Proc. Natl. Acad. Sci. USA., 107, 21931–21936. 21106759 10.1073/pnas.1016071107 PMC3003124

[18] GTex Consortium (2013) The genotype-tissue expression (GTEx) project. Nat. Genet., 45, 580–585. 23715323 10.1038/ng.2653 PMC4010069

[19] Klug M. and Rehli M. (2006) Funcitonal analysis of promoter CpG methylation using a CpG-free luciferase reporter vector. Epigenetics, 3, 127–130. 10.4161/epi.1.3.3327 17965610

[20] Banerjee C., Javed A., Choi J.Y., Green J., Rosen V., Wijnen A.J. van, Stein J.L., Lian J.B. and Stein G.S. (2001) Differential regulation of the two principal *Runx2*/*Cbfa1* n-terminal isoforms in response to bone morphogenetic protein-2 during development of the osteoblast phenotype. Endocrinology, 142, 4026–4039. 11517182 10.1210/endo.142.9.8367

[21] Enomoto H., Shiojiri S., Hoshi K., Furuichi T., Fukuyama R., Yoshida C.A., Kanatani N., Nakamura R., Mizuno A., Zanma A. et al. (2003) Induction of osteoclast differentiation by *Runx2* through receptor activator of nuclear factor-kappa B ligand (RANKL) and osteoprotegerin regulation and partial rescue of osteoclastogenesis in *Runx2*^−/−^ mice by RANKL transgene. J. Biol. Chem., 278, 23971–23977. 12697767 10.1074/jbc.M302457200

[22] Sudhakar S., Li Y., Katz M.S. and Elango N. (2001) Translational regulation is a control point in *RUNX2*/*Cbfa1* gene expression. Biochem. Biophys. Res. Commun., 289, 616–622. 11716520 10.1006/bbrc.2001.6033

[23] Greenlatt M.B., Ritter S.Y., Wright J., Tsang K., Hu D., Glimcher L.H. and Aliprantis A.O. (2013) NFATc1 and NFATc2 repress osteoarthritis. Proc. Natl. Acad. Sci. USA., 110, 19914–19919. 24248346 10.1073/pnas.1320036110 PMC3856808

[24] Otto F., Thornell A.P., Crompton T., Denzel A., Gilmour K.C., Rosewell I.R., Stamp G.W., Beddington R.S., Mundlos S., Olsen B.R. et al. (1997) *Cbfa1*, a candidate gene for cleidocranial dysplasia syndrome, is essential for osteoblast differentiation and bone development. Cell, 89, 765–771. 9182764 10.1016/s0092-8674(00)80259-7

[25] Wang X., Manner P.A., Horner A., Shum L., Tuan R.S. and Nuckolls G.H. (2004) Regulation of MMP-13 expression by *RUNX2* and *FGF2* in osteoarthritic cartilage. Osteoarthritis Cartilage, 12, 963–973. 15564063 10.1016/j.joca.2004.08.008

[26] Zhong L., Huang X., Karperien M. and Post J.N. (2016) Correlation between gene expression and osteoarthritis progression in human. Int. J. Mol. Sci., 17. 10.3390/ijms17071126 PMC4964500 27428952

[27] Kraan P.M. van der and Berg W.B. van den (2012) Chondrocyte hypertrophy and osteoarthritis: role in initiation and progression of cartilage degeneration? Osteoarthritis Cartilage, 20, 223–232. 22178514 10.1016/j.joca.2011.12.003

[28] Liao L., Zhang S., Gu J., Takarada T., Yoneda Y., Huang J., Zhao L., Oh C.D., Li J., Wang B. et al. (2017) Deletion of *Runx2* in articular chondrocytes decelerates the progression of DMM-induced osteoarthritis in adult mice. Sci. Rep., 7, 2371. 28539595 10.1038/s41598-017-02490-w PMC5443810

[29] Gee F., Rushton M.D., Loughlin J. and Reynard L.N. (2015) Correlation of the osteoarthritis susceptibility variants that map to chromosome 20q13 with an expression quantitative trait locus operating on *NCOA3* and with functional variation at the polymorphism rs116855380. Arthritis Rheumatol., 67, 2923–2932. 26211391 10.1002/art.39278 PMC4832313

[30] Wilkins J.M., Southam L., Price A.J., Mustafa Z., Carr A. and Loughlin J. (2007) Extreme context specificity in differential allelic expression. Hum. Mol. Genet., 16, 537–546. 17220169 10.1093/hmg/ddl488

[31] Shepherd C., Skelton A.J., Rushton M.D., Reynard L.N. and Loughlin J. (2015) Expression analysis of the osteoarthritis genetic susceptibility locus mapping to an intron of the MCF2L gene and marked by the polymorphism rs11842874. BMC Med. Genet., 16, 108. 26584642 10.1186/s12881-015-0254-2 PMC4653905

[32] Reynard L.N., Ratnayake M., Santibanez-Koref M. and Loughlin J. (2016) Functional characterization of the osteoarthritis susceptibility mapping to *CHST11* - a bioinformatics and molecular study. PLoS One, 11, e0159024. 10.1371/journal.pone.0159024 PMC4938163 27391021

[33] Teare M.D., Pinyakorn S., Heighway J. and Santibanez Koref M.F. (2011) Comparing methods for mapping cis acting polymorphisms using allelic expression ratios. PLoS One, 6, e28636. 10.1371/journal.pone.0028636 PMC3236754 22174852

[34] Kokenyesi R., Tan L., Robbins J.R. and Goldring M.B. (2000) Proteoglycan production by immortalized human chondrocyte cell lines cultured under conditions that promote expression of the differentiated phenotype. Arch. Biochem. Biophys., 383, 79–90. 11097179 10.1006/abbi.2000.2044

[35] Takahashi A., Andres M.C. de, Hashimoto K., Itoi E., Otero M., Goldring M.B. and Oreffo R.O.C. (2017) DNA methylation of the RUNX2 P1 promoter mediates MMP13 transcription in chondrocytes. Sci. Rep., 7, 7771. 28798419 10.1038/s41598-017-08418-8 PMC5552713

