# Supplementary material for: Identification of a novel, methylation-dependent, RUNX2 regulatory region associated with osteoarthritis risk
Source: Hum Mol Genet. 2018 Aug 10;27(19):3464–74. doi: 10.1093/hmg/ddy257 (PMC6140783; doi:10.1093/hmg/ddy257)
Supplement: Supplementary Data [file ddy257_supp.zip › Supplementary Figures.pdf]

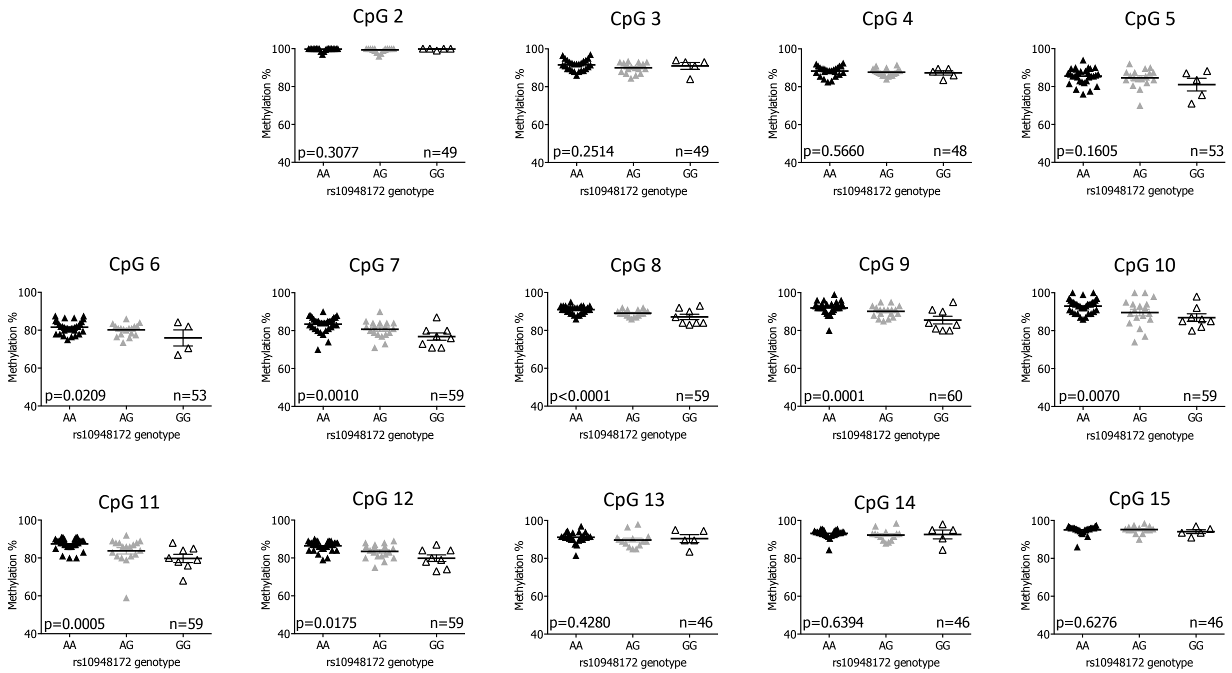

Supplementary Figure S1. Definition of the 5' and 3' limits of the *SUPT3H/RUNX2*-DMR in OA cartilage DNA. Graphs display the percentage methylation at each CpG site stratified by genotype at the OA association SNP rs10948172 (A/G). P values were calculated using a one-way analysis of variance (ANOVA) with a Bonferonni post-test correction. The horizontal line represents the mean methylation and the SEM. n, the number of patients analysed per CpG.

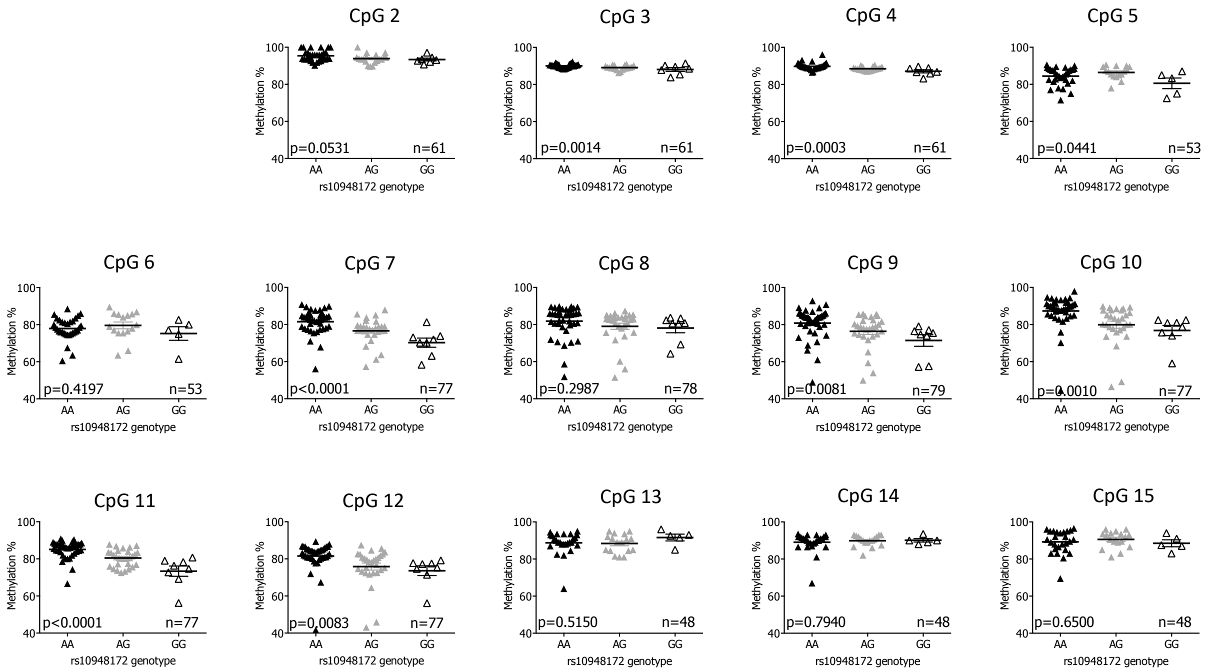

Supplementary Figure S2. Definition of the 5' and 3' limits of the *SUPT3H/RUNX2*-DMR in OA fat pad DNA. Graphs display the percentage methylation at each CpG site stratified by genotype at the OA association SNP rs10948172 (A/G). P values were calculated using a one-way analysis of variance (ANOVA) with a Bonferonni post-test correction. The horizontal line represents the mean methylation and the SEM. n, the number of patients analysed per CpG.

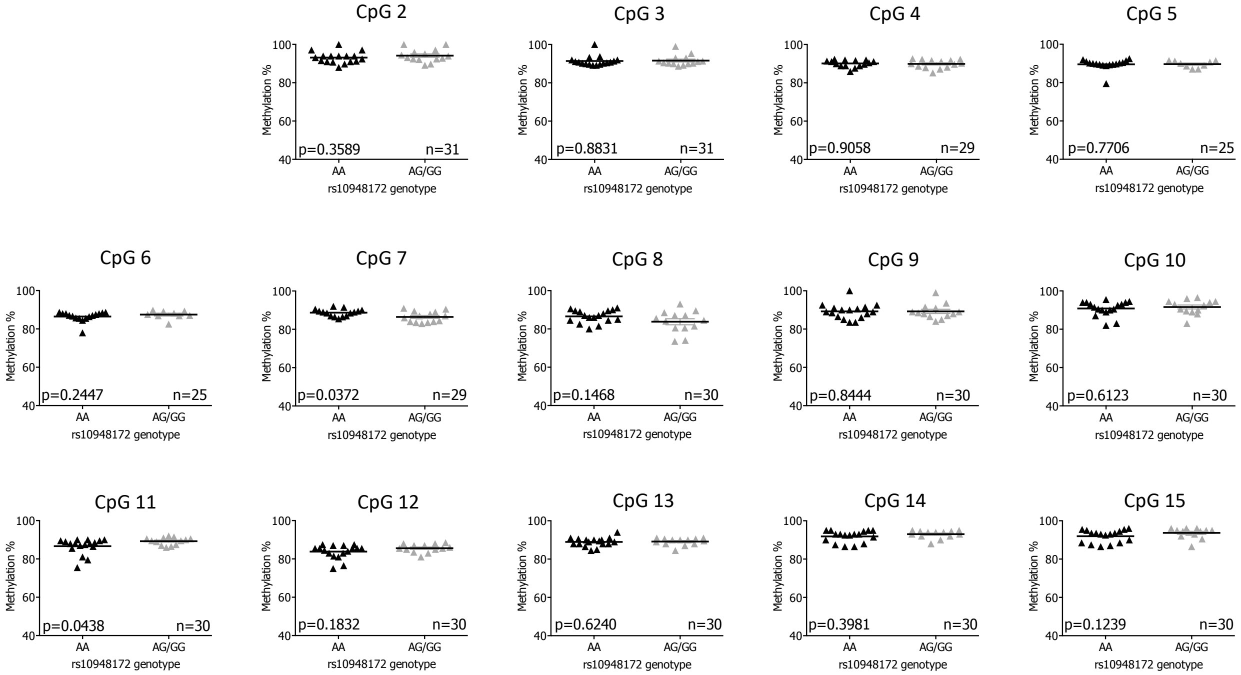

Supplementary Figure S3. Definition of the 5' and 3' limits of the *SUPT3H/RUNX2*-DMR in OA bone DNA. Graphs display the percentage methylation at each CpG site stratified by genotype at the OA association SNP rs10948172 (A/G). Due to the low number of homozygotes for the OA risk-conferring G allele, AG and GG genotypes were combined for the analysis. P values were calculated using a one-way analysis of variance (ANOVA) with a Bonferonni post-test correction. The horizontal line represents the mean methylation and the SEM. n, the number of patients analysed per CpG.

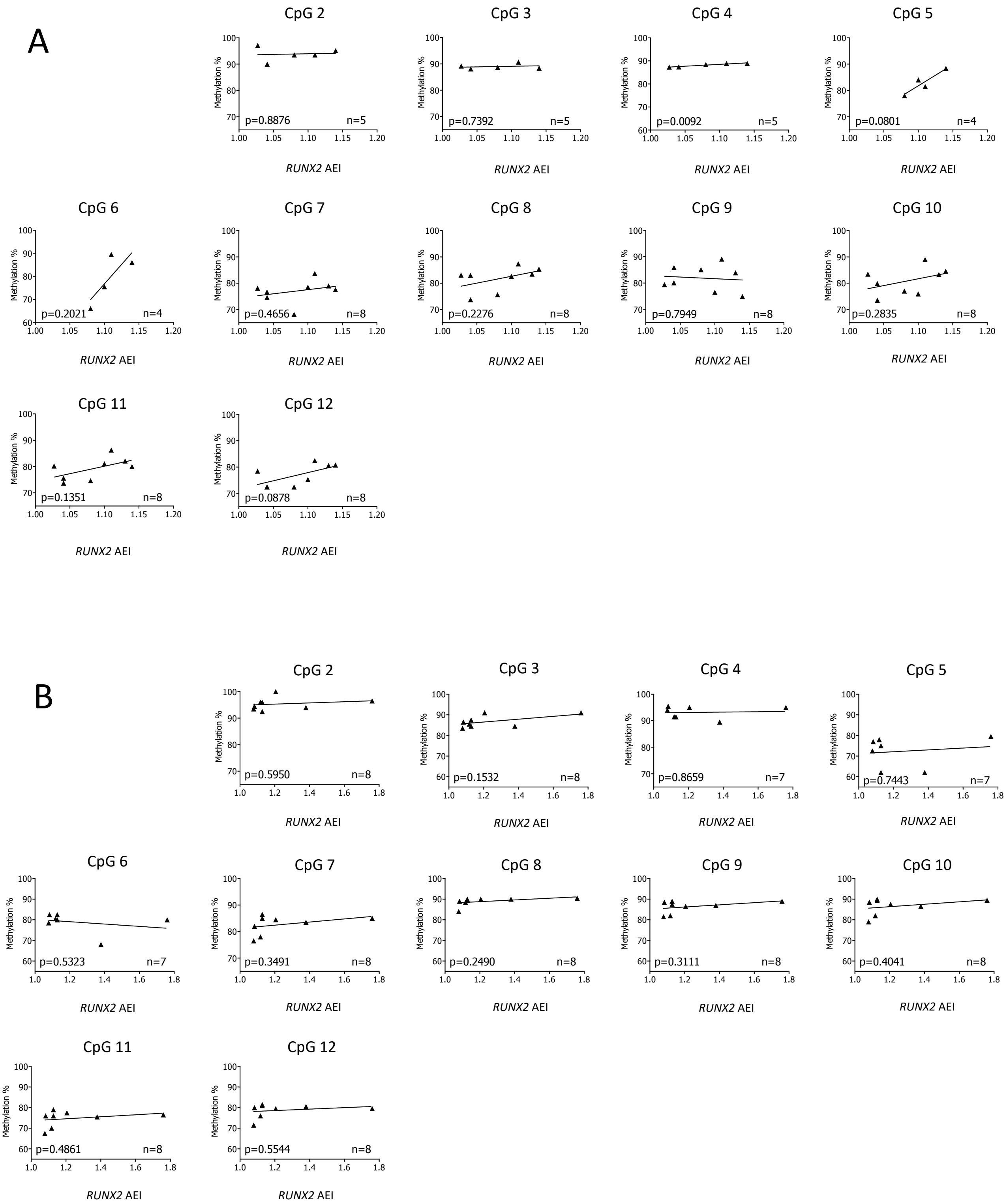

Supplementary Figure S4. Correlation between allelic expression imbalance (AEI) at *RUNX2* and methylation at the *SUPT3H/RUNX2*-DMR. Normalised cDNA allelic ratios of *RUNX2* expression in fat pad (A) and cartilage (B) were plotted against the respective DNA methylation values at CpG2 through to CpG12. P values were calculated by linear regression analysis. n, the number of patients analysed per CpG.

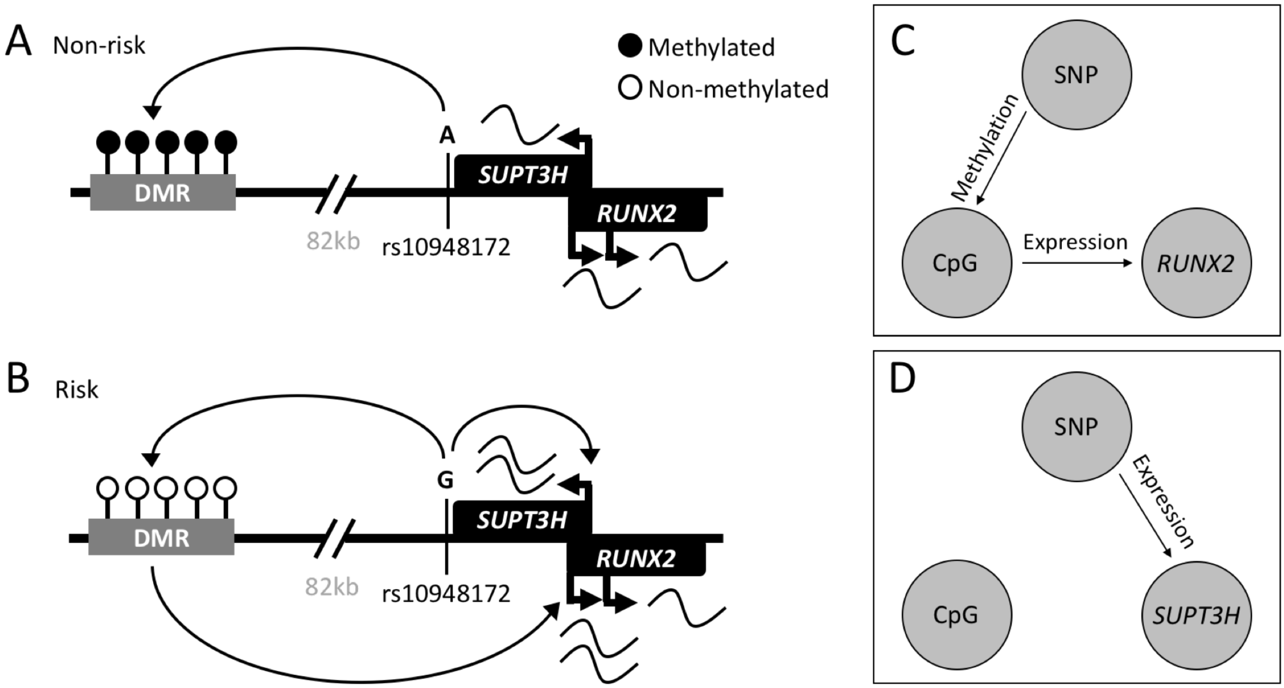

Supplementary Figure S5. Proposed mechanism of *RUNX2* regulation by the *SUPT3H/RUNX2*-DMR. A, The non-risk allele at rs10948172 (A) is associated with high levels of methylation at the DMR. **B,** The OA risk allele at rs10948172 (G) is associated with relatively lower levels of methylation at the DMR, and an alteration in the amount of *RUNX2* P1 transcript, compared with that measured in the presence of the A allele at rs10948172. The risk allele (G) is also associated with increased transcription of *SUPT3H.* However, this does not appear to be mediated by effects of methylation within the DMR. **C,** At this locus, genotype at the association SNP impacts upon methylation at CpG sites within the DMR, and regulates expression of the *RUNX2* P1 transcript. **D,** Genotype at the association SNP mediates regulation of *SUPT3H* expression, in a methylation-independent manner.

Patient ID	Sy	FP	Cn	Bn	rs10948172 Genotype	rs10948155 Genotype	rs62435998 Genotype	Sex	Age at Surgery (years)	Joint
1	Y				A G			F	71	K
2		Y			A A			F	42	K
3		Y			A A		C C	M	53	K
4		Y			A A			M	63	K
5		Y			A A	T T	C C	M	69	K
6		Y			A A	T T	C C	M	76	K
7		Y			A A	T T	C C	M	82	K
8	Y	Y			A A	T T	C C	F	55	K
9		Y			A G	T C	C T	F	68	K
10		Y			A G	T C	C T	M	63	K
11	Y				A A			M	81	K
12		Y			A A	T T	C C	F	65	K
13		Y			A G	T C	C T	M	66	K
14		Y			A A	T C	C T	F	74	K
15		Y			A G	T C	C T	F	86	K
16	Y		Y		G G	C C	T T	F	64	K
17	Y	Y			G G	C C	T T	F	78	K
18	Y				G G	C C	T T	F	65	K
19		Y			A G	T C	C T	M	63	K
20	Y				A A			M	69	K
21	Y				A G			F	72	K
22		Y			A G			M	74	K
23		Y			A A	T T	C C	F	69	K
24	Y				G G			F	64	K
25	Y	Y			A G	T C	C T	M	56	K
26		Y			A A	T T	C C	M	63	K
27		Y			A A	T T	C C	F	71	K
28	Y				A G			F	78	K
29	Y				A G	T C		M	56	K
30	Y				A G	T C	C T	F	54	K
31		Y			A G			F	62	K
32	Y				A A		C C	M	71	K
33		Y			A G	T C	C T	F	71	K
34	Y				G G	C C	T T	M	67	K
35		Y			A G	C C	C T	F	67	K
36		Y			A A	T T	C C	M	71	K
37			Y		A G			F	53	H
38		Y			G G			F	76	K
39	Y				A A			F	67	K
40		Y			A A	T T	C C	M	72	K
41		Y	Y		A G	T C		M	77	K
42		Y			A G	T C	C T	F	73	K
43	Y				A G	T C	C T	F	60	K
44		Y	Y		A A	T T	C C	M	78	K
45		Y			A G	C C	T T	M	56	K
46		Y			A A			M	67	K
47		Y			A G	T C	C T	M	65	K
48	Y				A G	C C	C T	M	85	K
49		Y			A G	T C	C T	M	70	K
50		Y			A A	T C	C T	F	55	K
51	Y				A A	T T	C C	F	79	K
52		Y			A A	T T	C C	F	48	K
53		Y			G G	C C	T T	M	49	K
54			Y		A A	T T	C C	M	67	K
55	Y				A A	T T	C C	M	65	K
56			Y		A A			M	68	K
57	Y				A G	T C	C T	F	72	K
58		Y			A G	T C	C T	F	61	K
59		Y			A A	T T	C C	F	76	K
60			Y		A G			F	72	K
61		Y			A G			F	60	K
62		Y			A A		C C	F	70	K
63		Y			A G	T C	C T	F	71	K
64		Y			A G			F	78	K
65		Y			A A	T C	C T	F	68	K
66		Y			A G			F	55	K
67			Y		A A	T T	C C	M	81	H
68			Y		A G			M	64	K
69	Y				A G	T C	C T	F	82	K
70		Y			A G			F	81	K
71		Y			A G	C C	C T	F	70	K
72			Y		G G	T C	T T	F	34	K
73		Y			A A	T C	C C	M	57	K
74		Y			G G	C C	T T	F	79	K
75		Y			A A		C C	F	78	K
76		Y			A A	T T	C C	F	77	K
77		Y			G G	T T	C C	M	69	K
78		Y			A A	T T	C C	F	62	K
79		Y			A A			M	69	K
80		Y			A A	T T	C C	M	67	K
81		Y	Y		A A	T T	C C	M	77	K
82		Y			A G			F	72	K
83		Y			A A	T T	C C	F	82	K
84			Y		A A	T C	C T	M	56	K
85			Y		A G	T C	C T	F	61	K
86			Y		A A	T C		F	73	H
87			Y		G G	C C	T T	M	58	H
88		Y			A A	T T	C C	F	65	K

89			Y		A A	T C	C C	F	66	K
90			Y		A G	T C	C T	F	78	K
91			Y		G G	C C		F	67	H
92	Y		Y		A G	T C	C T	M	51	K
93			Y		A G	T C	C T	F	45	H
94			Y		A G	T C	C T	F	67	H
95			Y		A G	T C	C T	F	45	H
96			Y		A A	T C	C T	F	68	K
97			Y		A A	T T		M	82	K
98			Y		A G	T C	C T	F	60	K
99			Y		A A	T T	C T	F	68	H
100			Y		A A	T T	C C	F	63	K
101			Y		A A	T T		F	87	K
102			Y		A A	T T	C C	M	59	K
103			Y		A A	T T	C C	F	69	K
104	Y				G G	T T	C C	M	75	K
105	Y				A A	T C	C T	M	62	K
106			Y		A A	T T		M	62	K
107			Y		A A	T C	C C	F	61	K
108			Y		A A	T T	C C	M	54	K
109			Y		A G	T C		F	57	K
110			Y		A A	T T	C C	M	55	K
111			Y		G G	C C	T T	M	50	K
112			Y		G G	C C	T T	M	50	K
113	Y		Y		A A	T T	C C	M	75	K
114			Y		A G	T C	C T	F	73	K
115			Y		A A	T T		F	61	K
116			Y		G G			M	82	K
117			Y		A A	T T	C C	F	73	K
118			Y		A A	T C		F	66	H
119			Y		A A			F	89	H
120			Y		A G	C C	T T	M	81	K
121			Y		G G	C C		M	65	H
122			Y		A A	T T	C C	F	52	H
123			Y		A A	T T	C C	F	64	K
124			Y		A G	T C	T T	M	74	K
125			Y		A G	T C		M	74	H
126			Y		A G	T C	C T	F	52	H
127			Y		A A	C C	T T	F	76	H
128			Y		A G	T T	C C	F	76	K
129	Y				A A	T T	C C	F	62	K
130	Y				A A	T T	C C	F	62	K
131	Y				A G	T C	C T	F	68	K
132	Y				A G	T C	C T	F	59	K
133			Y		A G	T C	C T	M	62	H
134	Y				A A	T T	C C	M	77	K
135			Y		A A	T T	C C	M	73	H
136	Y				A G	T C		F	75	K
137	Y				G G	T T	C C	M	87	K
138			Y		A A	T T	C C	F	70	K
139			Y		A A	T C	C T	F	61	H
140			Y		A A	T T		F	65	H
141	Y				A A	C C	T T	M	80	K
142	Y				A G	T T	C T	M	65	K
143	Y	Y			A A	T T	C C	M	72	K
144	Y		Y		A A	T T	C C	F	52	K
145	Y		Y		A A	C C	C C	F	62	K
146	Y				A G	T C	C T	M	67	K
147	Y	Y			G G	C C	T T	F	62	K
148	Y				A G	T C	C T	M	67	K
149	Y				A G	T C	C T	M	91	K
150	Y				A A	T T	C T	M	68	K
151	Y				G G	C C	T T	F	71	K
152	Y				A A	T T	C T	M	62	K
153	Y				A G			F	66	K
154	Y				A A	T C	C T	M	53	K
155	Y				A G	C C	C T	M	57	K
156	Y				A A	T C	C C	M	49	K
157	Y				A A	T T	C C	F	54	K
158			Y		A A	T T	C C	M	84	H
159	Y				A G	T C	C T	F	75	K
160	Y				A G	T C	C T	F	71	K
161		Y			A G	T C	C T	M	73	K
162	Y				A A			M	64	K
163	Y				A G	T C	C T	F	73	K
164	Y	Y			A G	T C	C T	F	63	K
165	Y				A A	T C		F	72	K
166	Y	Y			A A	T C	C T	M	61	K
167	Y	Y			A A	T T	C T	F	77	K
168	Y				A A	T T	C T	M	71	K
169	Y				A G	T C	C T	F	79	K
170	Y				A A	T C	C C	M	77	K
171	Y				A A	T T	C C	M	75	K
172	Y				A G	T T	T T	M	60	K
173	Y	Y			A A	T T	C C	F	61	K
174	Y				A A	T T	C C	F	52	K
175	Y				A G	T C	C T	M	76	K
176	Y				A G	T C	C T	M	76	K
177	Y				G G	T C	T T	F	66	K
178		Y			A G	T C	C T	F	73	K

179	Y				A G	T C	T T	F	82	K
180	Y				A G	C C	C T	M	85	K
181	Y				A G	T C	C T	M	61	K
182	Y				A A	T T	C C	F	73	K
183	Y				A A	T C	C C	M	60	K
184	Y				G G	C C	T T	M	54	K
185	Y				A A	T C	C T	F	70	K
186	Y				A A	T T	C C	F	51	K
187	Y				A A	T C	C C	F	66	K
188	Y				A A	T T	C C	F	81	K
189	Y	Y			A A	T T	C T	M	70	K
190			Y		A G			F	53	K
191			Y		A A			M	75	K
192			Y		A A			M	61	H
193			Y		G G			M	75	K
194			Y		A A			F	66	H
195			Y		A A			F	65	H
196				Y	A G	T C	C T	F	65	H
197				Y	A A	T T	C C	M	71	H
198			Y		A A			F	64	H
199				Y	A G	T C	C T	M	82	K
200				Y	A A	T T	C C	M	66	H
201				Y	A A	T T	C C	M	68	K
202				Y	A A	T T	C C	M	75	K
203				Y	A G	T C	C T	F	57	K
204			Y		A G			F	58	K
205				Y	A A	T T	C C	F	78	H
206			Y		A G			F	79	K
207				Y	A A	T C	C C	M	58	K
208				Y	A A	T T	C C	M	76	K
209				Y	A G	T T	C C	F	81	K
210				Y	A G	T C	C T	F	63	K
211				Y	A G	T C	C T	M	72	H
212			Y		A G			F	54	K
213				Y	G G	C C	T T	F	78	K
214				Y	A A	T T	C C	M	81	K
215				Y	A G	T C	C T	F	65	H
216				Y	A A	T T	C C	F	64	H
217				Y	A G	T C	C T	F	63	K
218				Y	A G	T C	C T	F	64	K
219				Y	A G	T C	C T	M	53	K
220				Y	A A	T T	C C	M	62	H
221				Y	A G	C C	C T	M	73	K
222				Y	A A	T T	C C	M	86	K
223				Y	G G	C C	T T	F	54	K
224				Y	A A	T T	C C	F	75	K
225				Y	A G	T C	C T	M	78	K
226				Y	A A	T T	C C	F	90	K
227				Y	A A	T T	C C	M	62	K
228				Y	A A	T T	C C	M	51	K
229				Y	A A	T T	C C	F	55	K
230				Y	A A	T T	C C	M	79	K
231		Y			A A		C C	F	65	K
232		Y			G G	C C	T T	M	68	K
233		Y			A G			M	58	K
234		Y			A A	T T		M	75	K
235		Y			G G			F	60	K
236		Y			A A	T T	C C	M	71	K
237		Y			A A	T T	C C	F	73	K
238	Y				A G	T T	C T	F	69	K
239		Y			A G		C T	F	82	K
240	Y				A G	T C	C T	M	72	K
241		Y			A A	T T	C C	F	62	K
242	Y				A A	T T	C C	M	69	K
243		Y			A A			F	88	K
244	Y	Y			A A	T T	C C	M	58	K
245	Y				A G			M	65	K
246			Y		A G			M	71	H
247			Y		A G			M	67	H
248			Y		A G			F	70	K
249			Y		A G			F	63	K
250			Y		A A			F	67	H
251			Y		G G			M	67	H
252			Y		A G			F	65	H
253			Y		A A			F	65	H
254			Y		A G			F	62	H
255			Y		A A			F	64	H
256			Y		A A			F	60	H
257			Y		A A			M	74	H
258			Y		A G			F	67	H
259			Y		A G			F	81	H
260			Y		A G			F	75	H

Table S1: Patient information and genotype data for the hip (H) and knee (K) OA patients in whom DNA from synovium (Sy), fat pad (FP), cartilage (Cn) and trabecular bone (Bn) was analysed.

SNP	Genotyping Method	5' Primer (5'-3')	3' Primer (5'-3')	Sequencing Primer (5'-3')	Enzyme
rs10948172/rs529125	Pyrosequencing	GAAGCAGGCATTGAAAATGAC	CTTCCCTTACACCACCAACTTTA	AAAATGGATAGCTGTCACTA	N/A
rs10948155	Pyrosequencing	TAGGATGTGGCTTTGGAATAGAC	CCTGAGGAACTCAAAGCATGTCT	ACTCAAAGCATGTCTCTG	N/A
rs62435998	RFLP	ATAGACGTATCCAGGGCTGTCATGGCC	CCCCCGAGAGGAGACAGCCAAAATACT	N/A	*HaeIII*

Table S2: Primer sequences used to genotype OA patient DNA samples.

Assay	Number of CpGs	Our CpG nomenclature	450k array nomenclature	Location (hg19)	Forward Primer (5'-3')	Reverse Preimer (5'-3')	Sequencing Primer (5'-3')
1	1	CpG1	n/a	chr6:44,694,974	GGTGGTTGAAAGAGGAAAAGTT	CCAAACCATAACAACCCTAAATAC	TGGTTGTGGGTTAGT
2	3	CpG2	n/a	chr6:44,695,195	AGTTGTATTTAGGGTTGTTATGGT	ACCTAACTACCACTATTACCAACCTATT	GTGAGATGGGATTTTTTT
		CpG3	n/a	chr6:44,695,203			
		CpG4	n/a	chr6:44,695,216			
3	2	CpG5	n/a	chr6:44,695,264	TAACTACCACTATTACCAACCTATTCAAAT	AGTTGTATTTAGGGTTGTTATGGT	TACCAACCTATTCAAATT
		CpG6	n/a	chr6:44,695,267			
4	1	CpG7	cg13979708	chr6:44,695,319	GGTTGGTAATAGTAGTAGTTAGGTTAGTGG	CCAAAATTCTACCCTACCTCCAACTCC	GTAGTTAGGTTAGTGGAAA
5	1	CpG8	cg19254793	chr6:44,695,348	AGGTTGGTAATAGTAGTAGTTAGGTTAGTG	AACAAATACCAACCCTCCCAAAATTCT	AAAATTATCCAAATCTTCCT
6	1	CpG9	cg20913747	chr6:44,695,428	AGGTTGGTAATAGTAGTAGTTAGGTTAGTG	AACAAATACCAACCCTCCCAAAATTCTAC	ATAAGATAGGAGTTGAGTATTATAA
7	3	CpG10	cg18551225	chr6:44,695,537	TTTTGGGAGGGTTAGTATTTGTTAAGTATG	CCTACTTCTTACCAACTCCTTCTAACTACC	TTGAAGTTAGAAAGTGAG
		CpG11	n/a	chr6:44,695,544			
		CpG12	n/a	chr6:44,695,547			
8	3	CpG13	n/a	chr6:44,695,767	GTGTTTGTAATTGGTAGTTAGAAGG	ACTCACCTTACCCTCCCAAATCCTA	CCTAACCTTCCTCCTATAA
		CpG14	n/a	chr6:44,695,779			
		CpG15	n/a	chr6:44,695,781			

Table S3: Primers used for pyrosequencing methylation analysis. The genomic coordinates of each CpG site captured by the assay are provided. CpGs 7-10 are the four positive CpGs from the 450k array analysis.

Patient ID	Phenotype	Sex	Age at Surgery (years)	Joint
A	OA	F	66	H
B	OA	F	76	H
C	OA	F	83	H
D	OA	F	78	H
E	OA	F	83	H
F	OA	F	60	H
G	OA	F	55	H
H	OA	F	82	H
I	OA	F	72	H
J	OA	F	72	H
K	NOF	F	85	H
L	NOF	F	71	H
M	NOF	F	81	H
N	NOF	F	72	H
O	NOF	F	84	H
P	NOF	F	94	H

Table S4: The 16 patients used for generating cartilage RNA-seq data.

Gene	Transcript SNP	Forward Primer (5'-3')	Reverse Preimer (5'-3')	Sequencing Primer (5'-3')
SUPT3H	rs529125	GAAGCAGGCATTGAAAATGAC	CTTCCCTTACACCACCAACTTTA	AAAATGGATAGCTGTCACTA
RUNX2	rs1200428	CACCATTAGGGACCATCTGTGATA	AACCTGGATTCTGGGCCAGT	GGCCAGTCCCTTTCC

Table S5: Primer sequences used to measure AEI by pyrosequencing.
