# Supplementary figures and images for: Identification of a novel, methylation-dependent, RUNX2 regulatory region associated with osteoarthritis risk
Source: Hum Mol Genet. 2018 Aug 10;27(19):3464–74. doi: 10.1093/hmg/ddy257 (PMC6140783; doi:10.1093/hmg/ddy257)

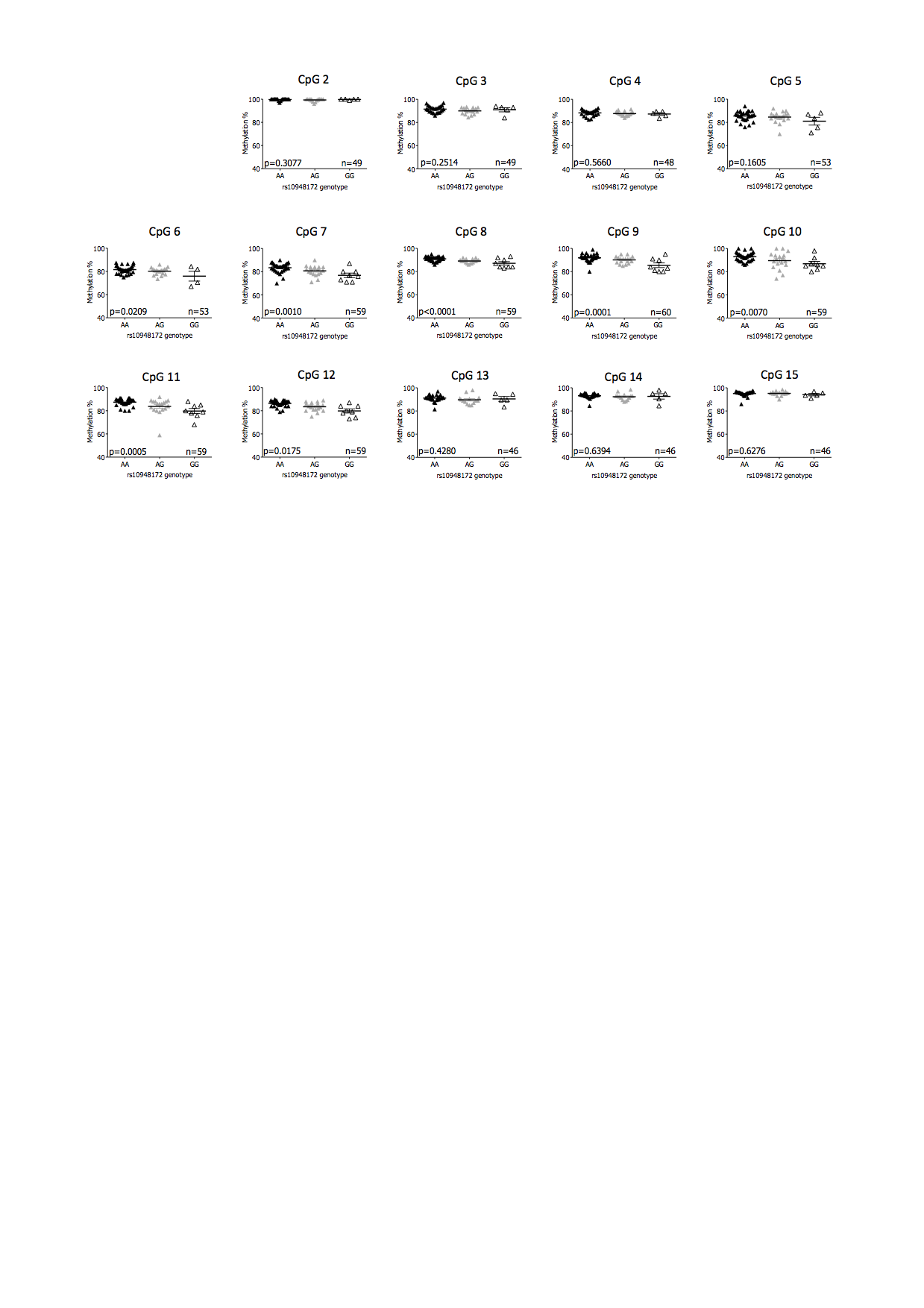

Supplement: Supplementary Data [file ddy257_supp.zip › Supplementary Figures.tiff]
